# Magnetic Properties of Electrospun Magnetic Nanofiber Mats after Stabilization and Carbonization

**DOI:** 10.3390/ma13071552

**Published:** 2020-03-27

**Authors:** Nadine Fokin, Timo Grothe, Al Mamun, Marah Trabelsi, Michaela Klöcker, Lilia Sabantina, Christoph Döpke, Tomasz Blachowicz, Andreas Hütten, Andrea Ehrmann

**Affiliations:** 1 Department of Physics, Center for Spinelectronic Materials and Devices, Bielefeld University, 33615 Bielefeld, Germany; nfokin@physik.uni-bielefeld.de (N.F.); andreas.huetten@uni-bielefeld.de (A.H.); 2 Faculty of Engineering and Mathematics, Bielefeld University of Applied Sciences, 33619 Bielefeld, Germany; timo.grothe@fh-bielefeld.de (T.G.); al.mamun@fh-bielefeld.de (A.M.); marah.trabelsi@enis.tn (M.T.); michaela.kloecker@fh-bielefeld.de (M.K.); lilia.sabantina@fh-bielefeld.de (L.S.); christoph.doepke@fh-bielefeld.de (C.D.); 3 Ecole Nationale d’Ingénieurs de Sfax (ENIS), Sfax 3038, Tunisia; 4 Institute of Physics–CSE, Silesian University of Technology, 44-100 Gliwice, Poland; tomasz.blachowicz@polsl.pl

**Keywords:** ferrimagnetic materials, superparamagnetism, magnetic hysteresis, magnetic materials, magnetic nanoparticles, nanocomposites, nanowires

## Abstract

Magnetic nanofibers are of great interest in basic research, as well as for possible applications in spintronics and neuromorphic computing. Here we report on the preparation of magnetic nanofiber mats by electrospinning polyacrylonitrile (PAN)/nanoparticle solutions, creating a network of arbitrarily oriented nanofibers with a high aspect ratio. Since PAN is a typical precursor for carbon, the magnetic nanofiber mats were stabilized and carbonized after electrospinning. The magnetic properties of nanofiber mats containing magnetite or nickel ferrite nanoparticles were found to depend on the nanoparticle diameters and the potential after-treatment, as compared with raw nanofiber mats. Micromagnetic simulations underlined the different properties of both magnetic materials. Atomic force microscopy and scanning electron microscopy images revealed nearly unchanged morphologies after stabilization without mechanical fixation, which is in strong contrast to pure PAN nanofiber mats. While carbonization at 500 °C left the morphology unaltered, as compared with the stabilized samples, stronger connections between adjacent fibers were formed during carbonization at 800 °C, which may be supportive of magnetic data transmission.

## 1. Introduction

Electrospinning makes it possible to create nanofiber mats from diverse materials, such as pure polymers [1,2,3], polymers blends, composite fibers from polymers and ceramics or metals [4,5], and cyclodextrins [6]. These can be used in diverse applications such as filters [7,8,9], biotechnology and tissue engineering [10,11,12], for energy harvesting and storage [13,14], or other “smart” functions [15]. Recently, a strong focus of diverse research groups has been related to electrospinning magnetic nanofibers, either as composites [16,17,18] or, after calcination of the composites to remove polymers, as pure metal nanofibers [19,20,21]. Such magnetic nanofiber mats can be used, e.g., as catalysts [22], for magnetic hyperthermia [23], or electromagnetic shielding [24]. In contrast to other methods, such as electrodeposition [25,26], seed-mediated growth [27], magnetic field patterning of magnetic precursor inks printed on a substrate [28], or electrochemical deposition [29], electrospinning has the advantage of enabling preparation of large-scale nanofiber networks in short times without the necessity to use a cleanroom, highly sophisticated equipment, or highly toxic material, and is thus often used to prepare magnetic nanofibers [30,31,32,33,34]. Notably, polyacrylonitrile (PAN) can be spun from the low-toxic solvent DMSO [3], making the whole process relatively easy to handle and avoiding unnecessary environmental pollution.

In contrast to other methods of producing magnetic nanofibers, electrospinning usually creates nanofibers with different bending radii and forms a nanofiber mat without or with only low-fiber orientation [35,36,37], resulting in much more complicated magnetic anisotropies than straight, even nanofibers [38,39,40]. This makes electrospun nanofiber mats challenging for some applications, e.g., in racetrack memory, which usually consists of an array of parallel arranged magnetic nanowires and can theoretically store data series in some ten- to hundred-domain walls per nanowire [41].

On the other hand, chaotic nanofiber mats or fiber networks with low orientation are interesting for other research areas; e.g., for bio-inspired neuromorphic computing [42]. This relatively new research area aims at reaching high-performance computing at low power consumption, avoiding the von Neumann bottleneck due to the separation of processor and memory in modern computers built according to the von Neumann architecture by integrating both parts, i.e., storing and calculating data in a structure inspired by the human brain. Recently, many attempts have been reported to implement neural networks, imitating the biological function of the brain, with conventional computers [43,44,45]. Much better performance in terms of speed and reduced power consumption, however, can be expected if physical networks of connected components are created to enable massive parallel computing [46,47,48,49].

Quasistatic and dynamic studies of the magnetic properties of bent nanofibers have been performed by different research groups, using simulations or experimental investigations which have underlined the aforementioned influence of diameter and curvature distributions [50,51,52]. For example, Alejos et al. showed that a local longitudinal field could be used to control the current-induced magnetization reversal in ferromagnetic strips. They used two bit-lines, above and below a ferromagnetic line sandwiched between a heavy metal and an oxidized layer. In such a heavy metal/ferromagnet/oxide triple layer, current-induced switching due to a global in-plane field is well-known [53,54,55], while the local field created domain walls at defined positions—which is necessary for racetrack applications [52].

For potential application in neuromorphic computing, memristors or other elements are often integrated in statistic fiber networks [56,57,58], an approach which cannot be realized with a single-step electrospinning process. Here, instead, we report on a combination of magnetic nanofibers with beads, tailored by a reduction of the polymer content in the spinning solution, as evaluated in detail in a former study [59]. This combination of long, thin nanofibers with beads along the fibers allows for combining data processing and storage, as shown in former studies [18,60,61,62,63].

## 2. Materials and Methods

### 2.1. Electrospinning

For the spinning solution, 14% polyacrylonitrile (PAN) (X-PAN, Dralon, Dormagen, Germany) was dissolved in dimethyl sulfoxide (DMSO, min 99.9%, purchased from S3 chemicals, Bad Oeynhausen, Germany) by stirring at room temperature for 2 h with a magnetic stirrer. PAN was chosen due to the possibility of stabilizing and carbonizing it after electrospinning, making it conductive [64].

To make the fibers magnetic, nanoparticles from Fe_3_O_4_ (magnetite, particle size 50–100 nm) and Fe_2_O_3_/NiO (diiron nickel tetraoxide or nickel ferrite, particle size <50 nm) were added (both purchased from Merck KGaA, Darmstadt, Germany) by stirring manually for 10 min and dispersing the nanoparticles in an ultrasonic bath for 40 min at 35 °C at a frequency of 37 kHz. Both materials are ferrimagnetic in bulk form and become superparamagnetic for very small nanoparticles [65,66]. The polymer: nanoparticle weight ratio used here was 1:1.8, identical to the highest nanoparticle concentration used in a previous study [18].

The needleless electrospinning machine Nanospider Lab (Elmarco, Liberec, Czech Republic) was used to prepare nanofiber mats on a polypropylene nonwoven substrate. Electrospinning was performed using a high voltage of 80 kV, a nozzle diameter of 0.9 mm, a carriage speed of 150 mm/s, a ground-substrate distance of 240 mm, and an electrode-substrate distance of 50 mm. The temperature in the chamber was held at 22 °C, while the relative humidity was set to 32%.

### 2.2. Stabilization and Carbonization

Parts of the samples were afterwards stabilized in a B150 muffle furnace (Nabertherm, Germany), approaching a temperature of 280 °C with a heating rate of 0.5 K/min, followed by isothermal treatment for 1 h. This step was necessary to enable carbonization, since pure PAN is not thermally stable at temperatures far above 300 °C, while carefully stabilized PAN is not degraded by high temperatures. Subsequent carbonization was performed in a CTF 12/TZF 12 furnace (Carbolite Gero Ltd., Neuhausen, Germany), approaching temperatures of 500 and 800 °C, respectively, with a heating rate of 10 K/min in a nitrogen flow of 150 mL/min (STP), again followed by isothermal treatment for 1 h.

### 2.3. Experimental Investigations

Magnetic measurements were performed with an alternating gradient magnetometer (AGM) Princeton MicroMag (LakeShore Cryotronics, Westerville, OH, USA). The surface morphology of the nanofiber mats was investigated using a scanning electron microscope (SEM), the Zeiss 1450VPSE, and an atomic force microscope (AFM), the FlexAFM Axiom (Nanosurf, Liestal, Switzerland). For the chemical investigations, a Fourier-transform infrared (FTIR) spectrometer, Excalibur 3100 (Varian, Inc., Palo Alto, CA, USA), was used. Fiber diameters were evaluated using the software ImageJ 1.51j8 (from National Institutes of Health, Bethesda, MD, USA).

### 2.4. Simulations

For comparison with the experimental investigations of the magnetic properties, the micromagnetic simulation software OOMMF (Object-Oriented MicroMagnetic Framework) was used [67], applying finite differences for meshing and solving the Landau–Lifshitz–Gilbert equation of motion [68]. The following material parameters were chosen for Fe_3_O_4_ (Fe_2_O_3_/NiO) according to typical literature values [69,70,71,72,73]: M_S,magnetite_ = 500 × 10^3^ A/m (M_S,nickel-ferrite_ = 270 × 10^3^ A/m), exchange constant A_magnetite_ = 12 × 10^−12^ J/m (A_nickel-ferrite_ = 12 × 10^−12^ J/m), magneto-crystalline anisotropy constant K_1,magnetite_ = 11 × 10^3^ J/m^3^ (K_1,nickel-ferrite_ = −6.9 × 10^3^ J/m^3^). Since the electrospinning process can be expected to produce arbitrarily oriented crystallographic orientations of the nanoparticles, random orientations of the simulated grains of 5 nm diameter were chosen. While this approach would lead to strong modifications of the simulation results due to arbitrary crystallographic orientations in subsequent simulations, in case of cobalt or other materials with large magneto-crystalline anisotropy [74], here the influence of the shape anisotropy dominated the relatively small magneto-crystalline anisotropy, as usual in iron or permalloy samples of similar dimensions [75]. Setting the Gilbert damping constant to α = 0.5, a quasi-static case was modeled. Field sweeps were performed in the range of ±300 mT to reach saturation. 

**Figure 1:**
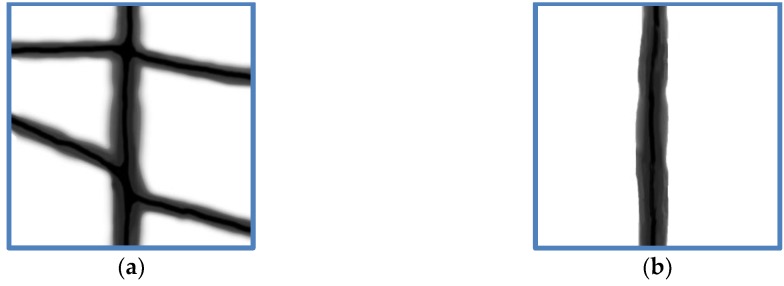
Simulated structures of dimensions 800 nm × 800 nm: (**a**) branched fiber; (**b**) single fiber.

The simulated shapes—a branched and a single fiber, respectively—were taken arbitrarily from an SEM image as an example of possible parts of such nanofiber mats (Figure 1). Fiber thickness was assumed as 120 nm, as according to the chosen part of a real magnetic nanofiber mat. Generally, the fiber thickness and thus the shape of the cross-section can be modified by a hot-pressing treatment [76]; this possibility was not further investigated here.

The simulated hysteresis loops shown here were superposed of 7 hysteresis loops each, simulated for external magnetic field sweeps in the range of ±3000 Oe with the field axis applied along 0° (horizontal in Figure 1), 15°, … 90° (vertical in Figure 1). This was equivalent to averaging over different nanofiber orientations in a constant external magnetic field, as it was applied during the AGM measurements on the magnetic nanofiber mats.

## 3. Results and Discussion

### 3.1. Morphological Investigations

**Figure 2:**
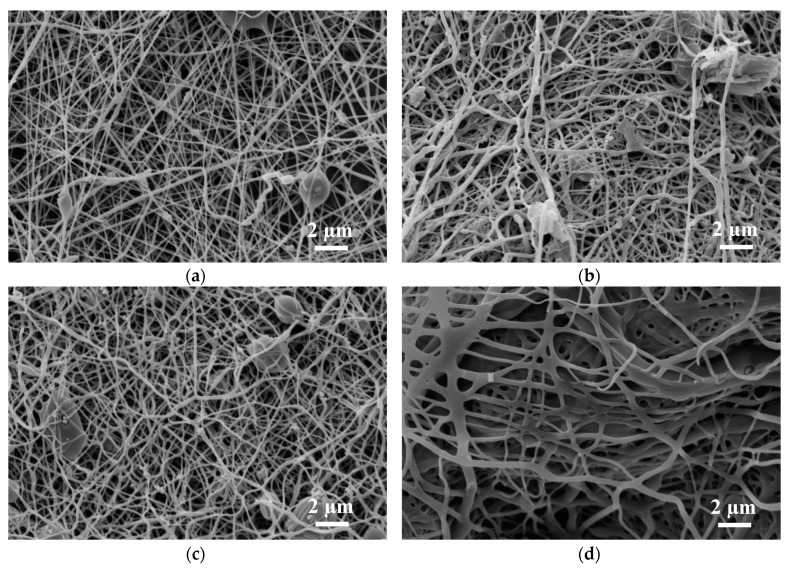
SEM images of polyacrylonitrile (PAN)/magnetite nanofiber mats: (**a**) after electrospinning; (**b**) after stabilization; (**c**) after carbonization at 500 °C; (**d**) after carbonization at 800 °C.

**Figure 3:**
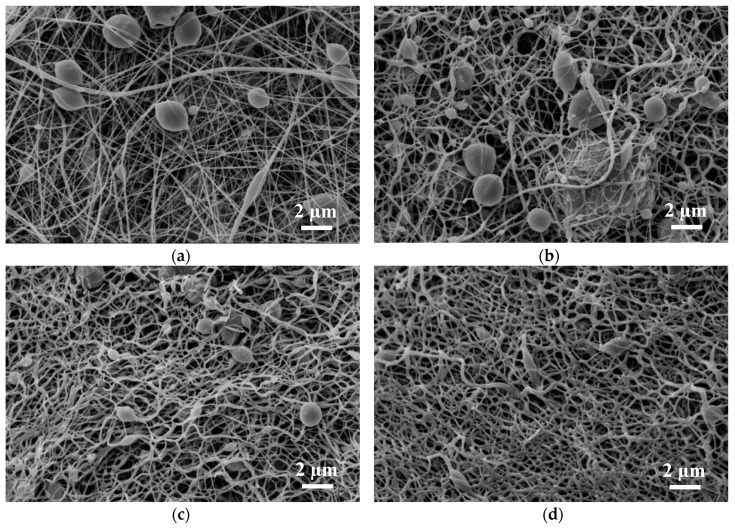
SEM images of PAN/nickel-ferrite nanofiber mats: (**a**) after electrospinning; (**b**) after stabilization; (**c**) after carbonization at 500 °C; (**d**) after carbonization at 800 °C.

Figure 2 depicts SEM images of the magnetite samples under examination; Figure 3 shows SEM images of the nickel-ferrite samples. As mentioned in Section 2, magnetic nanofiber mats with beads along the fibers were produced to test the combination of structures for data storage and transport for possible application in neuromorphic computing [18].

It should be mentioned that the aim of our recent study was not optimization of the numbers and dimensions of the beads, but investigation of the magnetic properties of nanofiber mats directly after electrospinning, as well as after stabilization and carbonization at different temperatures. Based on recent results and corresponding micromagnetic simulations, a further morphological optimization of the bead dimensions and quantities will be carried out.

In both nanofiber mats depicted in Figure 2 and Figure 3, the typical increase of the fiber diameter is visible due to relaxation of the internal stress in the nanofibers caused by the severe stretching during electrospinning [77]. Since the nanofiber mats were not mechanically fixed or even actively stretched during stabilization, this behavior can be expected, as discussed in detail in [77].

**Figure 4:**
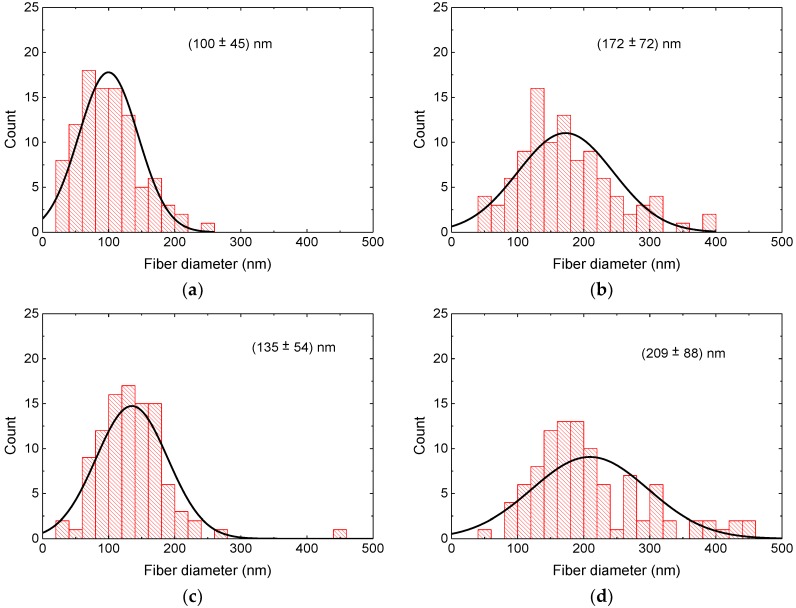
Distributions of the diameters of PAN/magnetite nanofibers: (**a**) after electrospinning; (**b**) after stabilization; (**c**) after carbonization at 500 °C; (**d**) after carbonization at 800 °C.

**Figure 5:**
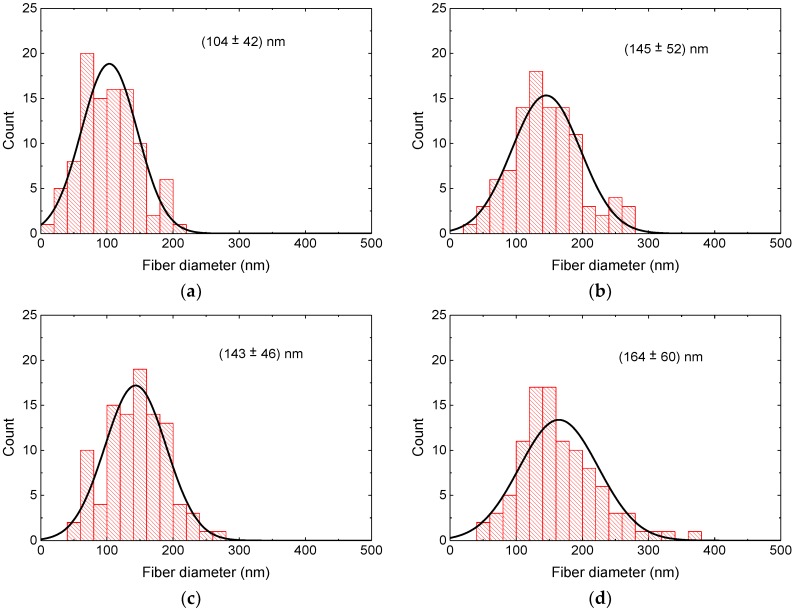
Distributions of the diameters of PAN/nickel-ferrite nanofibers: (**a**) after electrospinning; (**b**) after stabilization; (**c**) after carbonization at 500 °C; (**d**) after carbonization at 800 °C.

The diameter distributions of the nanofibers after the different treatments are depicted in Figure 4 and Figure 5. In PAN/magnetite as well as PAN/nickel-ferrite nanofiber mats, stabilization resulted in a larger average as well as in a broader distribution of the nanofiber diameters, the latter especially in case of PAN/magnetite. While the average diameter of PAN/magnetite nanofibers after carbonization at 500 °C was again smaller than after stabilization, both values were nearly identical for PAN/nickel-ferrite. Carbonization at 800 °C resulted in both material blends in the largest diameters. 

It should be mentioned, nevertheless, that these differences are not statistically significant due to the broad distributions of the measured fiber diameters and may partly be based on the arbitrary choice of the sample areas under investigation.

Both magnetic nanofiber mats showed the desired beads after electrospinning, with a larger number of relatively large beads in the case of PAN/nickel-ferrite. Future micromagnetic simulations are necessary to investigate which bead sizes are advantageous for data storage or use as switches, as shown in [18]. The fibrous structure of the beads stayed similar after stabilization due to the slow heating process, which was optimized in former studies for pure PAN nanofibers and PAN with integrated metal-oxide nanoparticles [78,79]. After carbonization at 500 °C, however, the beads in the PAN/nickel-ferrite nanofiber mats became smaller, an effect which has to be investigated in detail in the future. At 800 °C, the fiber structure of the PAN/magnetite nanofiber mats changed unexpectedly, which was not the case for PAN/TiO_2_ nanofiber mats [79] or PAN/gelatin nanofiber mats [80]. While this pure nanofiber structure is of great interest for data transport in magnetic networks, it cannot be used any more for date storage. Similarly, the beads were nearly lost in the PAN/nickel-ferrite nanofiber mat carbonized at 800 °C.

Apparently, it is necessary to either find a compromise between conductivity—which usually increases with increasing carbonization temperature and would support current-driven domain-wall transport—and the beads which could be used for data storage and manipulation. On the other hand, the carbonization parameters used here, especially the heating rate, were optimized for PAN, PAN/gelatin, and PAN/TiO_2_, and can possibly be further optimized in the cases of PAN/magnetite, and PAN/nickel-ferrite, respectively, to maintain the desired structure.

### 3.2. Chemical Investigations

**Figure 6:**
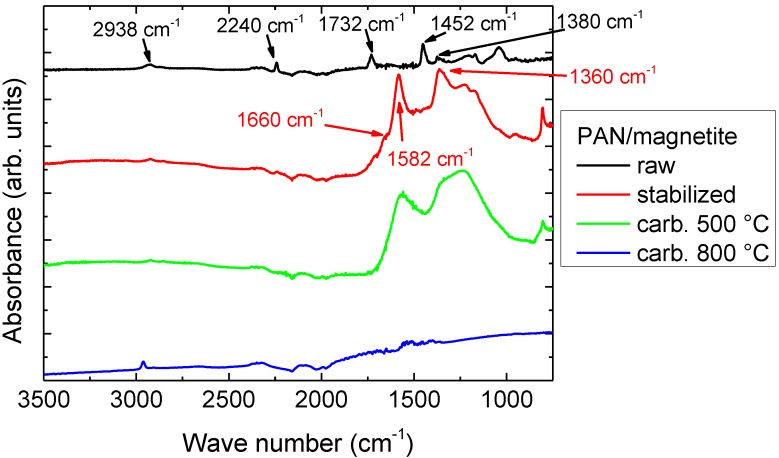
FTIR measurements of PAN/magnetite samples after electrospinning, stabilization, and carbonization at 500 and 800 °C. The lines are vertically offset for clarity.

Next, Figure 6 depicts FTIR spectra of PAN/magnetite samples; spectra measured for PAN/nickel-ferrite are approximately identical and are thus not shown here.

As described in detail in previous studies [78,79,80], the raw PAN/magnetite nanofiber mat was characterized by stretching vibrations of the C≡N nitrile functional group at 2240 cm^−1^, the carbonyl (C=O) stretching peak at 1732 cm^−1^, and bending and stretching vibrations of CH_2_ visible at 2938 cm^−1^, 1452 cm^−1^, and 1380 cm^−1^. In the raw nanofiber mat, no residues of the solvent DMSO were visible, underlining that only very small amounts of DMSO were left in these nanofiber mats.

In the stabilized nanofiber mats, the most prominent peaks were those of C=N stretching vibrations at 1582 cm^−1^, C=C stretching vibrations at 1660 cm^−1^, and C-H bending and C-H_2_ wagging around 1360 cm^−1^. After carbonization at 800 °C, the peaks nearly fully vanished since few functional groups were left after carbonization, resulting in the typical high absorbance of carbon. Temperature treatment at 500 °C was apparently not sufficient for a full carbonization process; instead, the FTIR spectrum still looked very similar to the stabilized sample.

Comparing the FTIR measurements with those performed on pure PAN nanofiber mats or PAN blends with gelatin, TiO_2_, etc. [78,79,80], no significant difference was visible due to the embedded magnetic nanoparticles.

### 3.3. Magnetic Investigations

**Figure 7:**
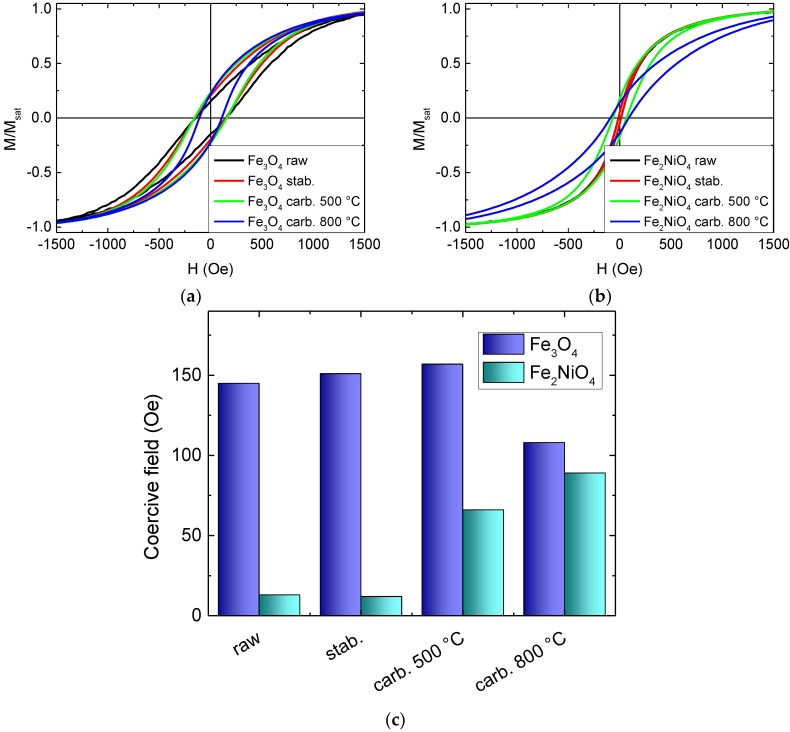
Alternating gradient magnetometer (AGM) measurements of hysteresis loops, performed on magnetic nanofibers including nanoparticles from (**a**) magnetite; (**b**) nickel-ferrite; and (**c**) coercive fields measured on these samples.

The magnetic characteristics of the magnetic nanofibers with magnetite and nickel-ferrite nanoparticles, respectively, are depicted in Figure 7.

Firstly, it is clearly visible that the coercive fields of the magnetite-based nanofibers were significantly larger than those of the nickel-ferrite-based nanofibers. For the PAN/magnetite nanofiber mats, the coercive fields did not change strongly with temperature treatments, while the PAN/nickel-ferrite nanofiber mats showed a soft magnetic behavior with only a very small coercive field at low temperature and much larger coercive fields after carbonization processes. Since both materials have similar magnetic properties, this difference can be attributed to the different nanoparticle dimensions—while the magnetite nanoparticles with diameters of 50–100 nm were large enough to show ferrimagnetic characteristics even without agglomerations, the nickel-ferrite nanoparticles with diameters below 50 nm were partly small enough for superparamagnetic behavior. Only after carbonization, when the nanofibers started forming conglutinations, were the neighboring nickel-ferrite nanoparticles near enough to overcome the superparamagnetic limit.

It must be mentioned that the highest carbonization temperature of 800 °C was above the Curie temperatures of both magnetic materials, which is approx. 570 °C for both bulk samples and 517 °C [81] or only 495 °C [82] for nanocrystalline nickel-ferrite. Similarly, in Fe_3_O_4_ nanocrystals, strongly reduced Curie temperatures down to 440 °C were reported [83]. In addition, a cation reordering was reported to occur in magnetite above 427 °C, corresponding to a gradual transformation from disordered to partially ordered configuration of magnetite [84]. These structural and magnetic effects may also have influenced the hysteresis loop shapes, in addition to the strong mass and volume loss; the latter resulting from an overproportioned shortening of the fibers as compared to the diameter increase, depicted in Figure 4 and Figure 5, of the fibers during stabilization and carbonization [78,79,80], which automatically reduced the distances between neighboring nanoparticles and thus influenced their collective magnetic characteristics [85].

**Figure 8:**
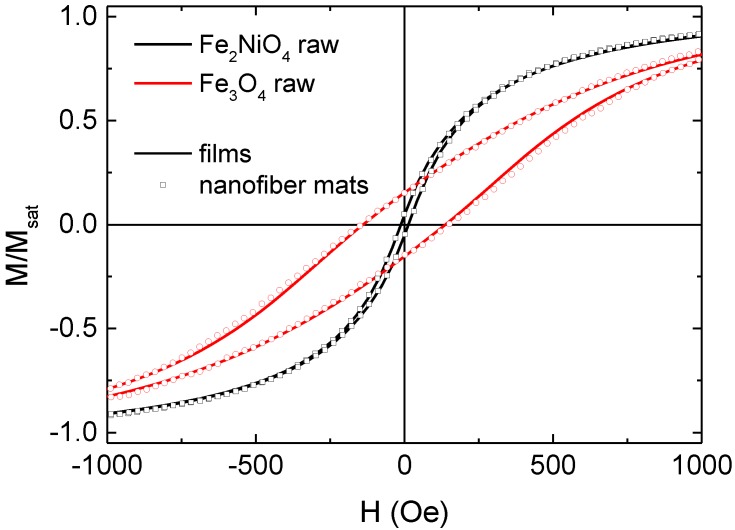
Hysteresis loops of as-prepared nanofiber mats and films from PAN with magnetite or nickel-ferrite nanoparticles, respectively.

Figure 8 shows a comparison of nanofiber mats and thin films, prepared from PAN with both magnetic nanoparticles used in this study. It is clearly visible that the magnetic properties were quite similar for both shapes, indicating that in both cases the nanoparticles were not near enough to each other to be subject to the magnetic shape anisotropy of the nanofibers, but were dominated by the single particle magnetic shape anisotropy. Whether there was any magnetic anisotropy of the particles inside the fibers, induced by electrospinning, has to be examined in future magnetic force microscope (MFM) investigations.

### 3.4. Micromagnetic Simulations

**Figure 9:**
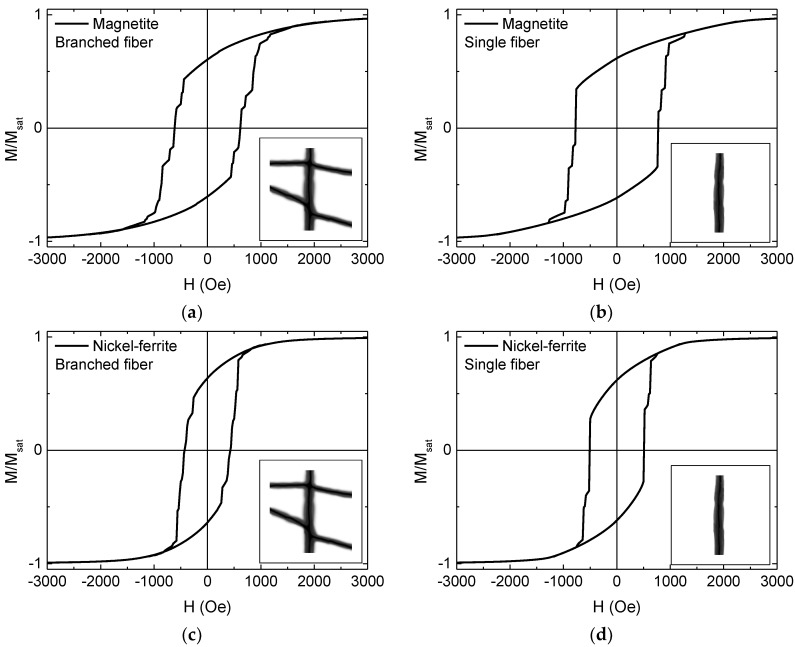
Simulated hysteresis loops of (**a**) magnetite branched fiber; (**b**) magnetite single fiber; (**c**) nickel-ferrite branched fiber; (**d**) nickel-ferrite single fiber.

This finding is underlined by the micromagnetic simulations depicted in Figure 9, showing hysteresis loops averaged over simulations along 0°, 15°, … 90° for magnetite and nickel-ferrite branched and single fibers, in this way simulating the expected results of macroscopic measurements on stochastically distributed pure magnetic nanofibers with or without branches.

The finding that coercive fields of nickel-ferrite nanofibers were generally smaller than those of magnetite nanofibers corresponds to the experimental results. The absolute values, however, were significantly larger in the simulation, underlining that the magnetic nanofibers prepared by electrospinning contained distributed magnetic nanoparticles which showed different magnetization reversal processes than the completely magnetic simulated nanoparticles. Apparently, simulations of stochastically distributed nanoparticle networks in the nanofiber shells are necessary to investigate in more detail the influence of inter-particle distances and the overall fiber shell, building a matrix in which the magnetic nanoparticles are distributed, on static and dynamic magnetization characteristics of the nanofiber network.

## 4. Conclusions

Magnetic nanofibers were prepared by electrospinning PAN nanofibers filled with magnetite or nickel-ferrite nanoparticles. While raw and stabilized fibers were relatively even and straight, more and more conglutinations between neighboring fibers occurred with increasing carbonization temperature, in case of magnetite leading to a deformation of the fiber shape from round fibers into flat ribbons. The magnetic properties were, especially in case of the smaller nickel-ferrite nanoparticles, strongly influenced by the thermal post-treatments, indicating that magnetization reversal is dominated by single-particle characteristics rather than by clusters of strongly interacting nanoparticles. Comparison with micromagnetic simulations of full-metal oxide nanofibers underlined this finding. It should be mentioned that care must be taken in the evaluation of magnetic properties after heating the nanofiber mats above the Curie temperatures of the magnetic nanoparticles; these thermal influences may have caused modifications of the magnetic properties in addition to the well-known structural changes of the nanoparticles due to a high-temperature treatment.

Future simulations of randomly distributed nanoparticles of different sizes inside a nanofiber matrix are necessary to fully understand the magnetic behavior of electrospun, stabilized, and carbonized magnetic nanofibers. To tailor research according to the necessities of data storage and transport for neuromorphic computing and other novel applications, additional MFM investigations on magnetization reversal and dynamics in these random nanofiber networks are necessary to produce reliable neuromorphic computing systems.

Additionally, on the materials science side, tailoring of the dimensions and quantities of the beads according to the afore-simulated necessities should be carried out, as well as an investigation of the stabilization and carbonization process in more detail, including examination of high-resolution images of the inner structures of the fibers by transmission electron microscopy (TEM). In this study, the optimum stabilization and carbonization parameters for pure electrospun PAN nanofibers were applied; however, it cannot be excluded that due to the embedded metallic nanoparticles, a new optimization study is necessary to avoid the unexpected formation of ribbon-like structures in the PAN/magnetite nanofiber mats after carbonization at 800 °C.
